# Effects of dietary β-mannanase supplementation in low-energy and low-protein diets containing high-mannan ingredients on growth performance, meat quality, intestinal morphology, and liver health in broiler chickens

**DOI:** 10.5713/ab.250545

**Published:** 2025-10-28

**Authors:** Eun Cheol Lee, Kang Hyeon Kim, Charline Mugeniwayesu, Ye Jin Jeong, Dong Yong Kil

**Affiliations:** 1 Department of Animal Science and Technology, Chung-Ang University, Anseong, Korea

**Keywords:** β-mannanase, Broiler Chicken, High-mannan Ingredient, Intestinal Morphology, Low-energy and Low-protein Diet, Performance

## Abstract

**Objective:**

This study was conducted to investigate the effect of dietary β-mannanase (MN) supplementation in low-energy and low-protein diets containing high-mannan ingredients on growth performance, meat quality, intestinal morphology, and liver health in broiler chickens.

**Methods:**

A total of 1,120 1-d-old Ross 308 broiler chickens were allotted to 1 of 5 dietary treatments. The positive control (PC) diet was prepared with corn and soybean meal, whereas the negative control (NC) diet was formulated with decreased AME_n_ by 100 kcal/kg and CP by approximately 1.0% than PC diets. High-mannan NC diet was also prepared by inclusion of 2.5% palm kernel meal (PKM) and 2.5% copra meal (CM), which contained energy and nutrient concentrations equal to those in the NC diet. Finally, dietary MN was supplemented to the high-mannan NC diet at 2 different levels of 0.05% and 0.10%.

**Results:**

Broiler performance was impaired (p<0.05) by feeding low-energy and low-protein diets, regardless of inclusion of PKM and CM. No positive effects of dietary MN supplementation in low-energy and low-protein diets containing PKM and CM on broiler performance were observed. Inclusion of PKM and CM increased (p<0.05) jejunal digesta viscosity but 0.10% MN supplementation decreased (p<0.05) jejunal digesta viscosity. Likewise, a tendency (p = 0.056) for increased villus height:crypt depth ratio was observed by 0.05% or 0.10% MN supplementation. However, dietary treatments had no effects on liver health in broiler chickens.

**Conclusion:**

Feeding low-energy and low-protein diets with reduction in 100 kcal/kg AME_n_ and approximately 1.0% CP impaired broiler performance. Inclusion of 2.5% PKM and 2.5% CM with or without MN supplementation had no considerable effects on broiler performance. However, MN supplementation in low-energy and low-protein diets containing PKM and CM decreased digesta viscosity with improving jejunal morphology.

## INTRODUCTION

The livestock feed industry is currently encountering challenges associated with rising feed costs, which have been primarily driven by the increasing demand for grains and oilseeds across various markets. In particular, the demand for corn and soybean meal, which serve as primary feed ingredients in poultry diets, is steadily escalating owing to the global population growth and the subsequent rise in poultry product consumption. Therefore, formulating cost-effective poultry diets that maintain productive performance and product quality in poultry is increasingly challenging. To address these challenges, poultry nutritionists have attempted to reduce energy and nutrient concentrations in diets with exogenous enzyme supplementation [1,2] and to increase the incorporation of economical alternative ingredients [3].

Palm kernel meal (PKM) and copra meal (CM) have been considered alternative ingredients for animal feeds because of their high nutritional value and relatively low cost. Research indicates that inclusion of PKM or CM at levels up to 10% in poultry diets can sustain poultry performance [4–6] with decreasing feed costs [7]. Nonetheless, poultry nutritionist remains cautious about including PKM and CM in poultry diets, primarily because these ingredients are rich in non-starch polysaccharides (NSPs), particularly β-mannan, which is regarded as a significant antinutritional factor that impairs nutrient digestion and utilization in the gastrointestinal tract (GIT) of poultry, leading to a reduction in productive performance and health in poultry [8–10]. In response to these challenges, dietary β-mannanase (MN), an exogenous enzyme that hydrolyzes dietary β-mannan, has frequently been supplemented in poultry diets containing high-mannan ingredients such as PKM and CM. Dietary MN supplementation in poultry diets containing high-mannan ingredients has shown comparable effects on productive performance and product quality in poultry relative to those observed with feeding conventional corn-soybean meal-based diets [11,12]. Moreover, dietary MN supplementation has been reported to enhance energy and nutrient utilization in poultry diets by reducing digesta viscosity and improving intestinal morphology and health in poultry [13]. Therefore, energy and nutrient-saving effects in poultry diets may be expected by dietary MN supplementation [14–16]. However, the studies regarding the effect of dietary MN supplementation in low-energy and low-protein diets on growth performance, meat quality, intestinal health, and liver health in broiler chickens are largely lacking. Furthermore, to our knowledge, no previous studies have explored the effects of dietary MN supplementation in broiler diets containing high-mannan ingredients, including PKM and CM, within the context of low-energy and low-protein diets.

Therefore, the current study aimed to investigate the effect of dietary MN supplementation in low-energy and low-protein diets containing PKM and CM on productive performance, meat quality, intestinal morphology, digesta viscosity, and liver health in broiler chickens.

## MATERIALS AND METHODS

### Animals, diets, and experimental design

A total of 1,120 one-d-old Ross 308 male and female broiler chickens (initial body weight [BW]±standard deviation = 43.7±0.29 g) were raised in a conventional floor pen (200×230 cm; width×length). A two-phase feeding program was implemented, comprising a grower diet from 1 to 21 d and a finisher diet from 22 to 35 d. Within each phase, 3 treatment diets were formulated. The positive control (PC) diet mainly comprised corn, soybean meal, and corn gluten meal with energy and nutrient concentrations meeting or exceeding their recommendations for Ross 308 broiler chickens [17]. Specifically, PC diets for the grower phase contained 3,100 kcal/kg AME_n_ and 21.50% CP, whereas those for the finisher phase contained 3,200 kcal/kg AME_n_ and 19.50% CP. The negative control (NC) diet as a low-energy and low-protein diet was prepared with corn and soybean meal by reducing 100 kcal/kg AME_n_ (i.e., 3,000 and 3,100 kcal/kg AME_n_ for grower and finisher phases, respectively) and approximately 1.0% CP (i.e., 20.43% and 18.53% CP for grower and finisher phases, respectively) as compared to those in the PC diet. Although the reduction in CP levels appeared to be 1.0% numerically, but this reduction actually represented 5.0% decrease relative to total CP levels in the PC diet for each phase. An additional diet containing high amounts of β-mannan, referred to as high-mannan NC diet in this study, was formulated by inclusion of 2.5% PKM and 2.5% CM with a partial replacement of corn and soybean meal in the NC diet while maintaining equivalent energy and nutrient concentrations to those in the NC diet. Furthermore, dietary MN (CTCzyme; declared activity of 800,000 U/kg; CTCBIO) was supplemented to the high-mannan NC diet at 2 different levels of 0.05% and 0.10% in place of celite. All diets were prepared in a mash form. The nutritional compositions of the PKM and CM used in this study are presented in Table 1 [18], whereas those in treatment diets, including PC, NC, and high-mannan NC diets, are presented in Tables 2, 3.

All chicks were randomly allotted to 28 floor pens with each housing 40 chicks (20 male and 20 female chicks). The PC and NC treatment groups were assigned to 10 replicated pens (n = 5), whereas the remaining 3 high-mannan treatment groups occupied the 18 pens (n = 6) due to limitations in our experimental facility. Diets and water were provided ad libitum for a 35-d feeding trial. The room temperature was maintained at 30°C during the first wk and was gradually decreased to 20°C by the end of the experiment. The average relative humidity throughout the study was 49.0±7.0%. The experiment was conducted under a continuous 24-h lighting scheme. The floor pens were furnished with fresh rice hulls as bedding material.

The body weight gain (BWG) and feed intake (FI) were recorded at the conclusion of the experiment, whereas mortality was monitored daily. After adjusting for mortality, the feed conversion ratio (FCR) was calculated by dividing FI by BWG [19].

### Sample collection and analysis

PKM and CM samples were finely ground for chemical analysis. Detailed procedures for determining the concentrations of dry matter, gross energy, nitrogen, ether extract, neutral detergent fiber (NDF), acid detergent fiber (ADF), and total amino acids in PKM and CM were presented in our previous study [18].

At the conclusion of the experiment (i.e., 35 d of age), one male chicken per replicate, with BW approximating the replicate mean, was selected and euthanized using CO_2_ asphyxiation. A blood sample was immediately collected from each bird via a heart puncture into a 10-mL EDTA-treated vacutainer tube for the plasma collection (Becton and Dickinson company, Diagnostics). This bird was also used to measure breast meat quality, jejunal morphology, digesta viscosity, and liver health.

### Breast meat quality

The breast muscle was excised for meat quality measurements and stored at 4°C until further analysis. The right portion of the breast meat was divided into four sections, two of which were used to measure pH at 1 and 24 h postmortem using a pH meter (Hanna Instruments). The meat color was determined using a colorimeter based on the Commission Internationale de l’Eclairage (CIE) color scale (Minolta Chroma Meter CR-400; Konica Minolta) for measuring lightness (L*), redness (a*), and yellowness (b*). The remaining two portions were used to determine water holding capacity (WHC) at 24 h postmortem and thiobarbituric acid-reactive substance (TBARS) values after storage at 4°C for 7 d, following the methods outlined by Choi et al [20]. This analysis was performed at the BT Research Facility Center, Chung-Ang University.

### Intestinal morphology

The jejunal morphology was examined using the method described by Nari et al [21]. Briefly, jejunal samples were flushed and fixed at 10% buffered formalin solutions. Each jejunal fragment was subsequently embedded in paraffin. A 5-mm section from each paraffin-embedded sample was placed onto a glass slide, stained with hematoxylin and eosin, and examined under a light microscope. Villus height (VH), crypt depth (CD), villus width (VW), and the ratio of VH to CD (VH:CD) were determined by taking 20 measurements from each jejunal sample. The methodology was detailed in the previous study [22].

### Digesta viscosity

Digesta viscosity was determined following the method of Choct and Annison [23]. Briefly, digesta samples were centrifuged at 9,000×g at 4°C for 10 min to obtain the supernatant. The relative viscosity was measured using an Ostwald viscosimeter, which features a U-shaped tube containing two bulbs, two calibration marks, and a capillary bore in one arm. The centrifuged digesta sample was drawn into the upper bulb of the viscosimeter by suction, allowing it to flow through the capillary into the lower bulb. The two calibration marks, positioned one above and below the upper bulb, indicated a known volume. The time required for the fluid level to traverse between these marks was used to calculate the kinematic viscosity. The flow times for aliquots of the digesta supernatant and distilled water were recorded. The relative viscosity of each sample was determined by dividing the time for the digesta supernatant by the time for distilled water.

### Plasma measurements and liver health

Blood samples were collected and immediately centrifuged at 3,000×g at 4°C for 15 min to obtain the plasma. The supernatant was transferred to a 1.7-mL microtube (Axygen) and stored in a refrigerator at −20°C prior to further analyses. The plasma concentrations of alanine aminotransferase (ALT) and aspartate aminotransferase (AST), which serve as potential indicators of liver health, were analyzed using a Hitachi Automatic Analyzer 7020 (Hitachi).

Furthermore, the subjective evaluation of liver color was conducted using a scale from 1 to 5 (1 = dark red; 5 = yellowish red) to investigate the incidence of fatty liver development [24]. The criteria for fatty liver color scores were derived from a previous study [25]. These subjective measurements were performed by 5 investigators using a blind test. For the blind test, investigators received a preliminary training to assign liver color scores based on standardized reference liver photographs. Thereafter, the investigator independently evaluated the identical set of liver images without access to treatment information. The average scores from each investigator were subsequently used for data analysis. In addition, the CIE color scale for L*, a* and b* values in the liver were also measured using a colorimeter (model CR-10; Konica Minolta). Following imaging and dissection of the liver, the small pieces of the liver were excised from the middle portion of the liver. The liver tissue samples were then placed into a 1.7-mL microtube, immediately transferred to liquid nitrogen, and stored at −80°C for the analysis of total fat concentrations. Total fat concentrations in the liver were assessed using acid-hydrolyzed ether extraction [26].

### Statistical analysis

All data were analyzed in a completely randomized design and PROC MIXED procedure of SAS (SAS Institute) was used for data analysis because of different number of replications per treatment. Each replicate served as an experimental unit for all statistical analyses. Possible outliers were verified using the UNIVARIATE procedure of SAS; however, none were identified. The LSMEANS procedure was used to calculate treatment means and the PDIFF option was used to separate the means if treatment effects were significant. A probability of p<0.05 was considered significant, whereas 0.05≤p≤ 0.10 was considered a tendency.

## RESULTS

### Growth performance and meat quality

Birds fed NC or high-mannan NC diets, regardless of MN supplementation, showed less (p<0.05) BW and BWG but greater (p<0.05) FCR than those fed PC diets (Table 4). However, no differences in BW, BWG, and FCR were observed between NC and high-mannan NC treatments. Moreover, increasing MN supplementation in high-mannan NC diets exhibited no differences in BW, BWG, and FCR as compared to NC or high-mannan NC diets. No difference in FI was observed among treatments.

Breast meat quality, including postmortem pH, meat color, WHC, and TBARS, was not affected by dietary treatments although b* value in the breast meat for birds fed NC diets was greater (p<0.05) than for those fed high-mannan NC diets with or without MN supplementation (Table 5).

### Jejunal morphology and digesta viscosity

Dietary treatments did not influence VH and VW in the jejunum (Table 6). However, birds fed PC or high-mannan NC diets with 0.05% and 0.10% MN supplementation tended to have less (p = 0.093) CD compared with those fed NC or high-mannan NC diets without MN supplementation. This reduction in CD corresponded to a tendency (p = 0.056) for increased VH:CD ratio in birds fed PC or high-mannan NC diets with 0.05% and 0.10% MN supplementation.

Feeding NC or high-mannan NC diets without MN supplementation to broiler chickens showed greater (p<0.05) digesta viscosity in the jejunum than feeding PC diets. Increasing MN supplementation from 0.05% to 0.10% in high-mannan NC diets decreased (p<0.05) digesta viscosity in the jejunum, in particular with 0.10% MN supplementation in high-mannan NC diets showing the similar digesta viscosity to PC diets.

### Liver health indicator

Plasma concentrations of AST and ALT as liver health indicators were not affected by dietary treatments (Table 7). Likewise, the subjective liver color score and CIE color values for the L*, a* and b* were not affected by dietary treatments. Total fat concentrations in the liver were not different among treatments groups.

## DISCUSSION

The observation of impaired broiler performance by a reduction of 100 kcal/kg AME_n_ and approximately 1.0% CP in broiler diets fortified with essential amino acids may indicate that the current energy and CP levels in low-energy and low-protein diets used in this study are likely inadequate to maintain the optimal broiler performance. Previous studies have reported that a decrease in energy concentrations by 80–100 kcal/kg AME in diets negatively affected growth performance in broiler chickens [27,28]. However, it has been documented that reducing CP levels by up to 3.0% while maintaining recommended essential amino acid levels exert no negative impacts on broiler performance [29]. Therefore, decreased performance was observed in broiler chickens fed low-energy and low-protein diets in this study, which may result from 100 kcal/kg AME_n_ reduction in broiler diets. Notably, no negative outcomes were identified in broiler performance by feeding low-energy and low-protein diets containing 2.5% PKM and 2.5% CM compared with feeding low-energy and low-protein diets formulated with corn and soybean meal. This finding may suggest that 2.5% PKM and 2.5% CM can be safely included in low-energy and low-protein broiler diets, provided that essential amino acids are adequately supplemented. Interestingly, increasing MN supplementation in low-energy and low-protein diets containing PKM and CM had no positive effects on broiler performance in this study. This result contrasts with the previous studies reporting that MN supplementation in diets containing high-mannan ingredients improved broiler performance [9,30]. Moreover, it has also been reported that MN supplementation in low-energy and low-nutrient diets improved broiler performance by decreasing intestinal viscosity and enhancing nutrient utilization [14–16]. In the current study, we also observed decreased intestinal viscosity and improved intestinal morphology by increasing MN supplementation in low-energy and low-protein diets containing PKM and CM, which may lead to an expectation for improved broiler performance. However, such an improvement by dietary MN supplementation on broiler performance was not identified in this study, which aligns with the previous findings [6,31]. The reason for this inconsistent result remain unknown; however, it may be attributed to the variation in animals, diet compositions, β-mannan contents, and experimental conditions among studies.

Dietary concentrations of energy, protein, and amino acids are recognized as important factors affecting meat quality in broiler chickens [32]. However, no considerable effects of dietary treatments on breast meat quality were found in this study. This lack of impacts may be related to equalized essential amino acid concentrations among all treatment diets [29]. Moreover, it has been reported that maintaining appropriate energy-to-protein ratio in diets is more critical to determine meat quality in broiler chickens rather than the absolute levels of energy and protein in diets [33]. Therefore, it is likely that decreased energy concentrations accompanied with reducing CP levels in low-energy and low-protein diets used in this study may result in an appropriate energy-to-protein ratio in diets, which may explain no significant impacts on breast meat quality in the current study. Despite differences in b* values among treatments, most meat quality values observed in this study were likely placed in the normal range of typical breast meat quality in broiler chickens. This finding may suggest that the current levels of energy and protein reduction, inclusion of PKM and CM, and MN supplementation in diets containing PKM and CM, exert no adverse impacts on breast meat quality in broiler chickens.

Increasing intake of viscous NSPs, such as β-mannan, has been documented to increase digesta viscosity in broiler chickens [34–36]. This finding agrees with our observation that feeding high-mannan diets containing PKM and CM increased jejunal digesta viscosity. Moreover, we also found a linear reduction in jejunal digesta viscosity by increasing MN supplementation in high-mannan diets, particularly revealing that 0.10% MN supplementation resulted in digesta viscosity comparable to PC treatment. These result corroborate the previous research indicating that dietary MN supplementation mitigated the rise in digesta viscosity due to increasing intake of dietary β-mannan in broiler chickens [34,36,37]. This decreased digesta viscosity by dietary MN supplementation may contribute to our observation for increased VH:CD ratio in the jejunum of broiler chickens fed high-mannan diets supplemented with MN. This positive relationship has been attributed to a decrease in turnover rates of intestinal cells resulting from decreased digesta viscosity [13,38]. Furthermore, it has been suggested that increasing breakdown of intact β-mannan by dietary MN diminished immune-stimulating actions of β-mannan in the GIT, which may enhance energy and nutrient supply for intestinal cell proliferation [13,39]. Consequently, the observation for decreased digesta viscosity and improved intestinal morphology may underlie improved nutrient and energy utilization in diets, thereby improving broiler performance by dietary MN supplementation [13]. However, despite the positive impact of dietary MN on intestinal measurements in this study, we found no beneficial effects on broiler performance.

Liver health is crucial for optimal performance and well-being in broiler chickens. In the current study, broiler chickens were fed low-energy and low-protein diets by reduction of 100 kcal/kg AME_n_ and approximately 1.0% CP. However, this dietary regimen did not affect various indicators of liver health and fatty liver incidence. Likewise, no negative or positive effects were observed by inclusion of 2.5% PKM and 2.5% CM in the low-energy and low-protein diets, regardless of dietary MN supplementation. Therefore, this finding may suggest that the current levels of energy and protein reduction, inclusion of PKM and CM in diets, and dietary MN supplementation in diets containing PKM and CM, have no negative effects on liver health and fatty liver development in broiler chickens. However, it should be noted that the current study was conducted only for 35 d. Therefore, extending the experimental period may yield different results. Further research is warranted to explore additional factors inducing fatty liver development in broiler chickens.

## CONCLUSION

Feeding low-energy and low-protein diets, characterized by a reduction of 100 kcal/kg AME_n_ and approximately 1.0% CP, impaired growth performance in broiler chickens, despite other nutrient concentrations meeting or exceeding their recommended levels. Inclusion of 2.5% PKM and 2.5% CM as high-mannan ingredients in low-energy and low-protein diets with or without MN supplementation had no negative impacts on broiler performance. However, supplementation of 0.10% MN in low-energy and low-protein diets containing PKM and CM decreased digesta viscosity and indicated a potential increase in jejunal VH:CD ratio. Nevertheless, meat quality and liver health in broiler chickens were not influenced by the current levels of energy and protein reduction, inclusion of PKM and CM in diets, and dietary MN supplementation.

## Figures and Tables

**Table 1:** Analyzed nutrient concentrations of the palm kernel meal and copra meal used in this experiment (%, as-fed basis)

Items	Palm kernel meal (PKM)	Copra meal (CM)
Dry matter (%)	90.8	89.1
Gross energy (kcal/kg)	4,317	3,946
Crude protein (%)	16.20	21.26
Neutral detergent fiber (%)	53.84	48.97
Acid detergent fiber (%)	30.30	23.03
Ether extract (%)	5.77	3.00
Total amino acids (%)		
Trp	0.14	0.15
Arg	1.54	1.50
His	0.28	0.31
Ile	0.50	0.53
Leu	1.04	1.16
Lys	0.48	0.35
Met	0.39	0.33
Phe	0.63	0.74
Thr	0.49	0.56
Val	0.74	0.82
Ala	0.67	0.80
Asp	1.16	1.37
Cys	0.31	0.42
Glu	2.72	3.26
Gly	0.70	0.77
Pro	0.60	0.73
Ser	0.61	0.75
Tyr	0.29	0.32

Adapted from Kim et al [18] with CC-BY.

**Table 2:** Composition and nutrient concentrations of experimental diets for grower phase (1 to 21 d)

Items	Positive control (PC)	Negative control (NC)	High-mannan NC
Ingredients (%)			
Corn	58.15	61.72	56.29
Soybean meal, 45% CP	23.99	25.00	23.90
Corn gluten meal	8.15	5.28	5.23
Palm kernel meal	-	-	2.50
Copra meal	-	-	2.50
Soybean oil	3.67	2.01	3.59
Monodicalcium phosphate	1.69	1.68	1.67
Limestone	1.41	1.40	1.39
_L_-Lysine HCl (78.5%)	0.56	0.46	0.49
_DL_-Methionine (98.5%)	0.26	0.31	0.32
_L_-Threonine (98.5%)	0.15	0.18	0.19
_L_-Valine (98.5%)	0.04	0.08	0.08
_L_-Isoleucine (98.5%)	0.03	0.06	0.07
_L_-Arginine (98.5%)	0.18	0.18	0.15
_L_-Tryptophan (98.5%)	0.01	0.10	0.01
Celite	0.30	0.30	0.30
Salt	0.20	0.20	0.20
50% Choline	0.15	0.15	0.15
NaHCO_3_	0.20	0.20	0.20
K_2_CO_3_	0.27	0.18	0.17
Coccidiostat	0.10	0.10	0.10
Antioxidant	0.20	0.20	0.20
Vitamin premix^1)^	0.15	0.15	0.15
Mineral premix^2)^	0.15	0.15	0.15
Total	100.00	100.00	100.00
Energy and nutrient contents^3)^			
AME_n_ (kcal/kg)	3,100	3,000	3,000
CP (%)	21.50	20.43	20.43
Digestible Lys (%)	1.15	1.15	1.15
Digestible Met+Cys (%)	0.87	0.87	0.87
Digestible Met (%)	0.59	0.59	0.59
Digestible Thr (%)	0.77	0.77	0.77
Digestible Val (%)	0.87	0.87	0.87
Digestible Ile (%)	0.78	0.78	0.78
Digestible Arg (%)	1.23	1.23	1.23
Digestible Trp (%)	0.18	0.18	0.18
Total calcium (%)	0.87	0.87	0.87
Available phosphorus (%)	0.44	0.44	0.44
DEB (mEq/kg)	200.00	200.00	200.00
Analyzed nutrient contents			
Analyzed CP (%)	21.25	20.77	21.02

PC, corn-soybean meal-based diet with recommended energy and CP levels; NC, corn-soybean meal-based diets with low-energy and low-protein diets (decreased AME_n_ by 100 kcal/kg and CP by 1.07% than PC diets); High-mannan NC, low-energy and low-protein NC diets formulated with 2.5% palm kernel meal and 2.5% copra meal.

1) Provided per kg of the complete diet: vitamin A, 12,000 IU (retinyl acetate); vitamin D_3_, 4,000 IU; vitamin E, 80.0 mg; vitamin K_3_, 4.0 mg (menadione dimethylpyrimidinol); vitamin B_1_, 4.0 mg; vitamin B_2_, 10.0 mg; vitamin B_6_, 6.0 mg; vitamin B_12_, 20.0 μg; folic acid, 2.0 mg; biotin, 200 μg; niacin, 60 mg.

2) Provided per kg of the complete diet: iron, 60 mg (FeSO_4_); zinc, 100 mg (ZnSO_4_); manganese, 120 mg (MnO); copper, 16 mg (CuSO_4_); cobalt, 1,000 μg (CoSO_4_); selenium, 300 μg (Na_2_SeO_3_); iodine, 1.25 mg [Ca(IO_3_)_2_].

3) Calculated values from Centraal Veevoederbureau (CVB) [40].

**Table 3:** Composition and nutrient concentrations of experimental diets for finisher phase (22 to 35 d)

Items	Positive control (PC)	Negative control (NC)	High-mannan NC
Ingredients (%)			
Corn	61.59	65.58	60.23
Soybean meal, 45% CP	20.43	20.20	18.95
Corn gluten meal	7.44	5.32	5.36
Palm kernel meal	-	-	2.50
Copra meal	-	-	2.50
Soybean oil	4.94	3.09	4.64
Monodicalcium phosphate	1.52	1.51	1.50
Limestone	1.29	1.29	1.28
_L_-Lysine HCl (78.5%)	0.43	0.45	0.48
_DL_-Methionine (98.5%)	0.24	0.27	0.28
_L_-Threonine (98.5%)	0.13	0.16	0.17
_L_-Valine (98.5%)	0.02	0.06	0.07
_L_-Isoleucine (98.5%)	0.03	0.07	0.08
_L_-Arginine (98.5%)	0.16	0.19	0.16
_L_-Tryptophan (98.5%)	0.01	0.01	0.02
Celite	0.30	0.30	0.30
Salt	0.20	0.20	0.20
50% Choline	0.15	0.15	0.15
NaHCO_3_	0.20	0.20	0.20
K_2_CO_3_	0.34	0.34	0.33
Coccidiostat	0.10	0.10	0.10
Antioxidant	0.20	0.20	0.20
Vitamin premix^1)^	0.15	0.15	0.15
Mineral premix^2)^	0.15	0.15	0.15
Total	100.00	100.00	100.00
Energy and nutrient contents^3)^			
AME_n_ (kcal/kg)	3,200	3,100	3,100
CP (%)	19.50	18.53	18.53
Digestible Lys (%)	1.03	1.03	1.03
Digestible Met+Cys (%)	0.80	0.80	0.80
Digestible Met (%)	0.54	0.54	0.54
Digestible Thr (%)	0.69	0.69	0.69
Digestible Val (%)	0.78	0.78	0.78
Digestible Ile (%)	0.71	0.71	0.71
Digestible Arg (%)	1.10	1.10	1.10
Digestible Trp (%)	0.16	0.16	0.16
Total calcium (%)	0.79	0.79	0.79
Available phosphorus (%)	0.40	0.40	0.40
DEB (mEq/kg)	200.00	200.00	200.00
Analyzed nutrient contents			
Analyzed CP (%)	18.19	17.58	17.35

PC, corn-soybean meal-based diet with recommended energy and CP levels; NC, corn-soybean meal-based diets with low-energy and low-protein diets (decreased AME_n_ by 100 kcal/kg and CP by 0.97% than PC diets); High-mannan NC, low-energy and low-protein NC diets formulated with 2.5% palm kernel meal and 2.5% copra meal.

1) Provided per kg of the complete diet: vitamin A, 12,000 IU (retinyl acetate); vitamin D_3_, 4,000 IU; vitamin E, 80.0 mg; vitamin K_3_, 4.0 mg (menadione dimethylpyrimidinol); vitamin B_1_, 4.0 mg; vitamin B_2_, 10.0 mg; vitamin B_6_, 6.0 mg; vitamin B_12_, 20.0 μg; folic acid, 2.0 mg; biotin, 200 μg; niacin, 60 mg.

2) Provided per kg of the complete diet: iron, 60 mg (FeSO_4_); zinc, 100 mg (ZnSO_4_); manganese, 120 mg (MnO); copper, 16 mg (CuSO_4_); cobalt, 1,000 μg (CoSO_4_); selenium, 300 μg (Na_2_SeO_3_); iodine, 1.25 mg [Ca(IO_3_)_2_].

3) Calculated values from Centraal Veevoederbureau (CVB) [40].

**Table 4:** Effect of β-mannanase (MN) supplementation in low-energy and low-protein diets containing high-mannan ingredients on growth performance in broiler chickens

Item	Dietary treatments^1)^					SEM	p-value

	Positive control (PC)	Negative control (NC)	High-mannan NC				

			0% MN	0.05% MN	0.10% MN		
BW (g)	1,945^a^	1,809^b^	1,832^b^	1,761^b^	1,801^b^	38.0	0.015
BWG (g)	1,902^a^	1,765^b^	1,789^b^	1,717^b^	1,757^b^	38.0	0.015
FI (g)	2,991	2,926	2,911	2,886	2,903	51.3	0.565
FCR (g/g)	1.57^b^	1.66^a^	1.63^a^	1.68^a^	1.65^a^	0.020	0.011
Mortality (%)	1.00	2.00	0.83	2.08	1.67	0.918	0.780

Data are least squares means of 5 observations in PC and NC treatments and 6 observations in other treatments.

1) PC, corn-soybean meal-based diet with recommended energy and CP levels; NC, corn-soybean meal-based diets with low-energy and low-protein diets; High-mannan NC, low-energy and low-protein NC diets containing 2.5% palm kernel meal and 2.5% copra meal. Dietary MN was supplemented to high-mannan NC diets at the levels of 0.05% and 0.10%.

a,b Mean values with different letters significantly differ at p<0.05.

SEM, standard error of the mean; BW, body weight; BWG, body weight gain; FI, feed intake; FCR, feed conversion ratio.

**Table 5:** Effect of β-mannanase (MN) supplementation in low-energy and low-protein diets containing high-mannan ingredients on breast meat quality of broiler chickens

Item	Dietary treatments^1)^						SEM	p-value

		Positive control (PC)	Negative control (NC)	High-mannan NC				

				0% MN	0.05% MN	0.10% MN		
Meat color	L*	44.2	45.3	43.5	46.0	45.1	0.84	0.214
	a*	3.0	3.1	3.2	2.8	2.9	0.41	0.955
	b*	14.5^ab^	15.1^a^	12.3^b^	12.0^b^	12.7^b^	0.83	0.039
pH	1 h	6.07	6.10	6.04	5.98	5.96	0.078	0.604
	24 h	5.45	5.49	5.57	5.49	5.59	0.058	0.307
WHC (%)		75.2	71.0	72.3	72.5	72.2	1.75	0.551
TBARS		0.35	0.32	0.33	0.32	0.35	0.010	0.066

Data are least squares means of 5 observations in PC and NC treatments and 6 observations in other treatments.

1) PC, corn-soybean meal-based diet with recommended energy and CP levels; NC, corn-soybean meal-based diets with low-energy and low-protein diets; High-mannan NC, low-energy and low-protein NC diets containing 2.5% palm kernel meal and 2.5% copra meal. Dietary MN was supplemented to high-mannan NC diets at the levels of 0.05% and 0.10%.

a,b Mean values with different letters significantly differ at p<0.05.

SEM, standard error of the mean; L*, lightness; a*, redness; b*, yellowness; WHC, water holding capacity; TBARS, thiobarbituric acid-reactive substance.

**Table 6:** Effect of β-mannanase (MN) supplementation in low-energy and low-protein diets containing high-mannan ingredients on jejunal morphology and digesta viscosity in broiler chickens

Item	Dietary treatments^1)^					SEM	p-value

	Positive control (PC)	Negative control (NC)	High-mannan NC				

			0% MN	0.05% MN	0.10% MN		
Jejunal morphology							
VH (μm)	1,570	1,570	1,496	1,521	1,513	67.2	0.893
CD (μm)	166	224	222	188	179	17.9	0.093
VW (μm)	150	168	170	160	155	9.9	0.354
VH:CD	10.86	8.41	7.94	9.23	9.47	0.705	0.056
Digesta viscosity							
Viscosity (cPs)	1.48^b^	1.67^a^	1.65^a^	1.56^ab^	1.48^b^	0.044	0.006

Data are least squares means of 5 observations in PC and NC treatments and 6 observations in other treatments.

1) PC, corn-soybean meal-based diet with recommended energy and CP levels; NC, corn-soybean meal-based diets with low-energy and low-protein diets; High-mannan NC, low-energy and low-protein NC diets containing 2.5% palm kernel meal and 2.5% copra meal. Dietary MN was supplemented to high-mannan NC diets at the levels of 0.05% and 0.10%.

a,b Mean values with different letters significantly differ at p<0.05.

SEM, standard error of the mean; VH, villus height; CD, crypt depth; VW, villus width.

**Table 7:** Effect of β-mannanase (MN) supplementation in low-energy and low-protein diets containing high-mannan ingredients on liver health indicators in broiler chickens

Item	Dietary treatments^1)^						SEM	p-value

		Positive control (PC)	Negative control (NC)	High-mannan NC				

				0% MN	0.05% MN	0.10% MN		
Plasma measurements								
AST (U/L)		319	339	316	286	331	34.4	0.815
ALT (U/L)		2.27	1.99	2.30	2.93	2.90	0.361	0.184
Liver measurements								
Color	L^*^	27.0	25.9	26.6	26.2	24.2	0.78	0.100
	a^*^	16.9	14.3	15.3	15.3	14.4	0.95	0.315
	b^*^	9.1	7.0	7.4	7.5	7.5	0.72	0.278
	Score^2)^	1.32	1.24	1.03	1.27	1.03	0.122	0.267
Total fat concentration (% DM)		15.5	14.5	16.4	16.7	12.9	1.85	0.516

Data are least squares means of 5 observations in PC and NC treatments and 6 observations in other treatments.

1) PC, corn-soybean meal-based diet with recommended energy and CP levels; NC, corn-soybean meal-based diets with low-energy and low-protein diets; High-mannan NC, low-energy and low-protein NC diets containing 2.5% palm kernel meal and 2.5% copra meal. Dietary MN was supplemented to high-mannan NC diets at the levels of 0.05% and 0.10%.

2) Subjective liver color score from 1 to 5 (1 = dark red; 5 = yellowish red).

SEM, standard error of the mean; AST, aspartate aminotransferase; ALT, alanine aminotransferase.

## Data Availability

Upon reasonable request, the datasets of this study can be available from the corresponding author
